# Ultrasound-Assisted Catheter-Directed Thrombolysis in Pulmonary Embolism: A Case Series

**DOI:** 10.7759/cureus.81303

**Published:** 2025-03-27

**Authors:** Hisham H Khalil, Safwat Aboaly, Saleh Alkhalifah, Modhahir Almossabeh, Ibrahim H Alsayegh, Zuhair Al Sulaiman

**Affiliations:** 1 Cardiology, Almoosa Specialist Hospital, Al Mubarraz, SAU; 2 Pulmonology, Almoosa Specialist Hospital, Al Mubarraz, SAU; 3 Medicine, Almoosa Specialist Hospital, Al Mubarraz, SAU

**Keywords:** catheter-directed thrombolysis, pulmonary embolism, right ventricular dysfunction, thrombolytic therapy, ultrasound-assisted thrombolysis

## Abstract

Pulmonary embolism (PE) is a life-threatening condition associated with significant morbidity and mortality. While systemic thrombolysis is effective in high-risk cases, it carries a substantial risk of bleeding complications. Ultrasound-assisted catheter-directed thrombolysis (UCDT) has emerged as a promising alternative, enhancing clot resolution while minimizing hemorrhagic risks. This case series presents three patients with high-risk and intermediate-high-risk PE who underwent UCDT. All patients exhibited hemodynamic instability and right ventricular dysfunction. Following UCDT, significant clinical and echocardiographic improvements were observed, with no major bleeding complications. UCDT represents a targeted thrombolysis approach that optimizes clot dissolution while reducing systemic thrombolytic exposure. Current evidence supports UCDT as an effective and safer alternative for select PE patients, though further randomized studies are needed to refine patient selection and standardize treatment protocols. This case series highlights the clinical benefits of UCDT and reinforces its role as a viable treatment strategy.

## Introduction

Pulmonary embolism (PE) is a life-threatening condition that remains a major cause of cardiovascular morbidity and mortality worldwide [1]. The clinical presentation of PE varies widely, ranging from asymptomatic cases to life-threatening hemodynamic instability. Early diagnosis and appropriate management are crucial in preventing adverse outcomes [2]. Traditional treatment strategies involve systemic anticoagulation, which remains the cornerstone of therapy for most patients with PE. However, in cases of high-risk PE with hemodynamic compromise, systemic thrombolysis is often required [3].

Despite its efficacy, systemic thrombolysis is associated with a significant risk of major bleeding complications, including intracranial hemorrhage, which limits its widespread use in certain patient populations [4]. Consequently, there has been growing interest in catheter-directed therapies, particularly ultrasound-assisted catheter-directed thrombolysis (UCDT), which aims to enhance clot dissolution while reducing systemic bleeding risks [5]. UCDT employs ultrasound energy to facilitate deeper thrombolytic agent penetration into the clot, theoretically improving efficacy and allowing for lower doses of thrombolytics [6].

Recent studies, including the Ultrasound Accelerated Thrombolysis of Pulmonary Embolism (ULTIMA) trial, have demonstrated that UCDT results in the faster resolution of right ventricular (RV) dysfunction and improved hemodynamic stability compared to anticoagulation alone, with a more favorable safety profile than systemic thrombolysis [1]. Additionally, meta-analyses suggest that UCDT may provide a mortality benefit in high-risk and intermediate-high-risk PE patients while reducing the incidence of major bleeding complications [2]. This case series aims to contribute to the existing body of literature by presenting the outcomes of three patients with high-risk and intermediate-high-risk PE who were managed with UCDT.

## Case presentation

Case 1

A 55-year-old woman with no known history of cardiomyopathy, chronic pulmonary disease, or cancer underwent total knee replacement due to severe osteoarthritis. Three days postoperatively, she developed sudden shortness of breath and palpitations. Upon arrival at the emergency department, she was hemodynamically unstable with a blood pressure of 70/40 mmHg, an oxygen saturation of 88% on room air, tachycardia, and tachypnea.

The Wells score was calculated at 9, indicating a high probability of PE. Laboratory tests revealed elevated troponin (1.48 µg/L), B-type natriuretic peptide (BNP) (64.5 pmol/L), and D-dimer (3.32 µg/mL). Bedside transthoracic echocardiography (TTE) showed a moderately dilated RV, mildly reduced RV systolic function, and positive McConnell's sign, suggestive of acute PE.

**Figure 1:**
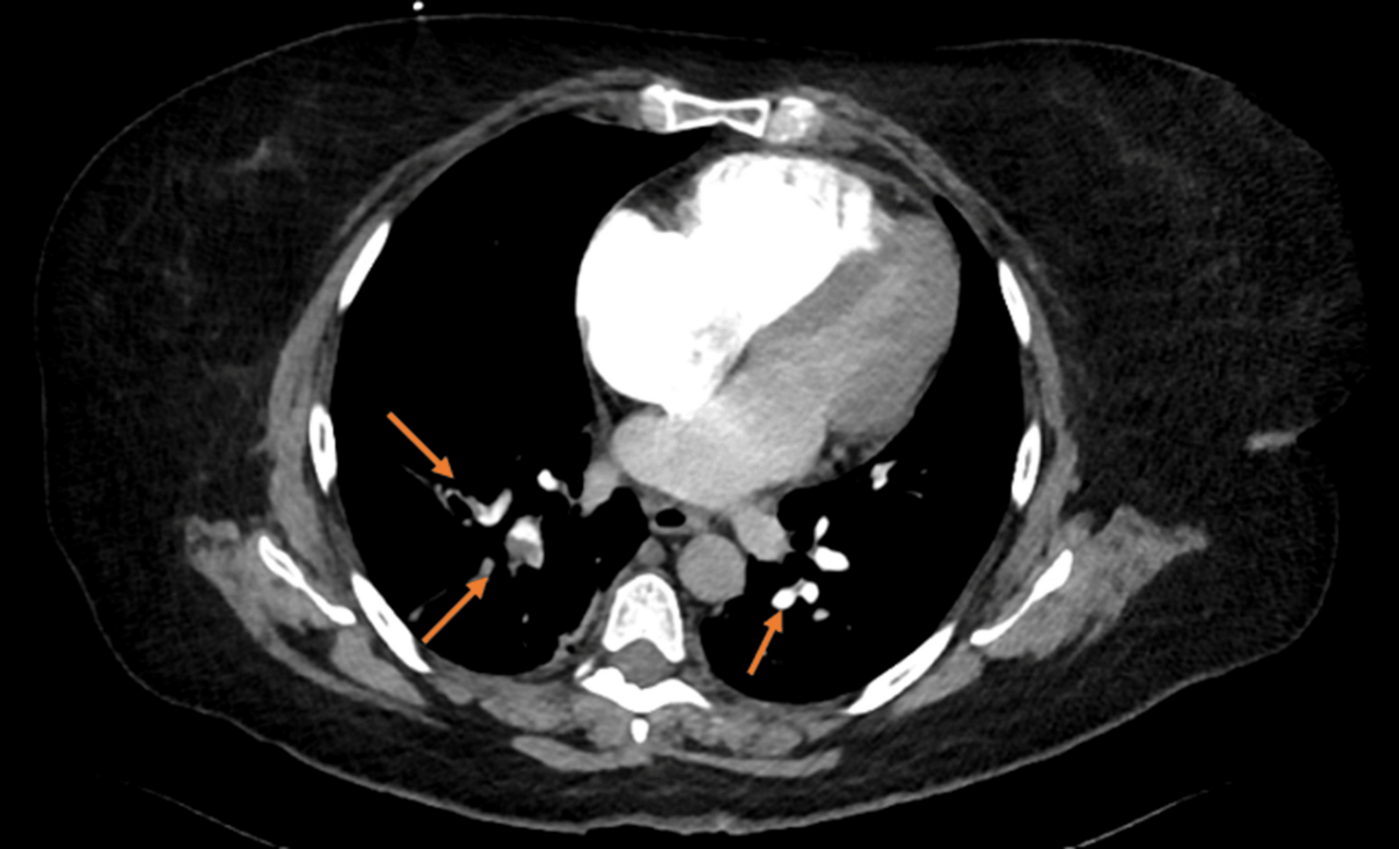
CTPA with orange arrows indicating multiple segmental and subsegmental pulmonary artery thrombosis CTPA: computed tomography pulmonary angiography

Urgent computed tomography angiography (CTA) confirmed acute pulmonary thromboembolism involving multiple segmental and subsegmental branches (Figure 1). Venous duplex ultrasonography showed no evidence of deep vein thrombosis (DVT). Given the high-risk PE classification based on the simplified Pulmonary Embolism Severity Index (PESI), the patient underwent UCDT. The procedure was uneventful.

**Figure 2:**
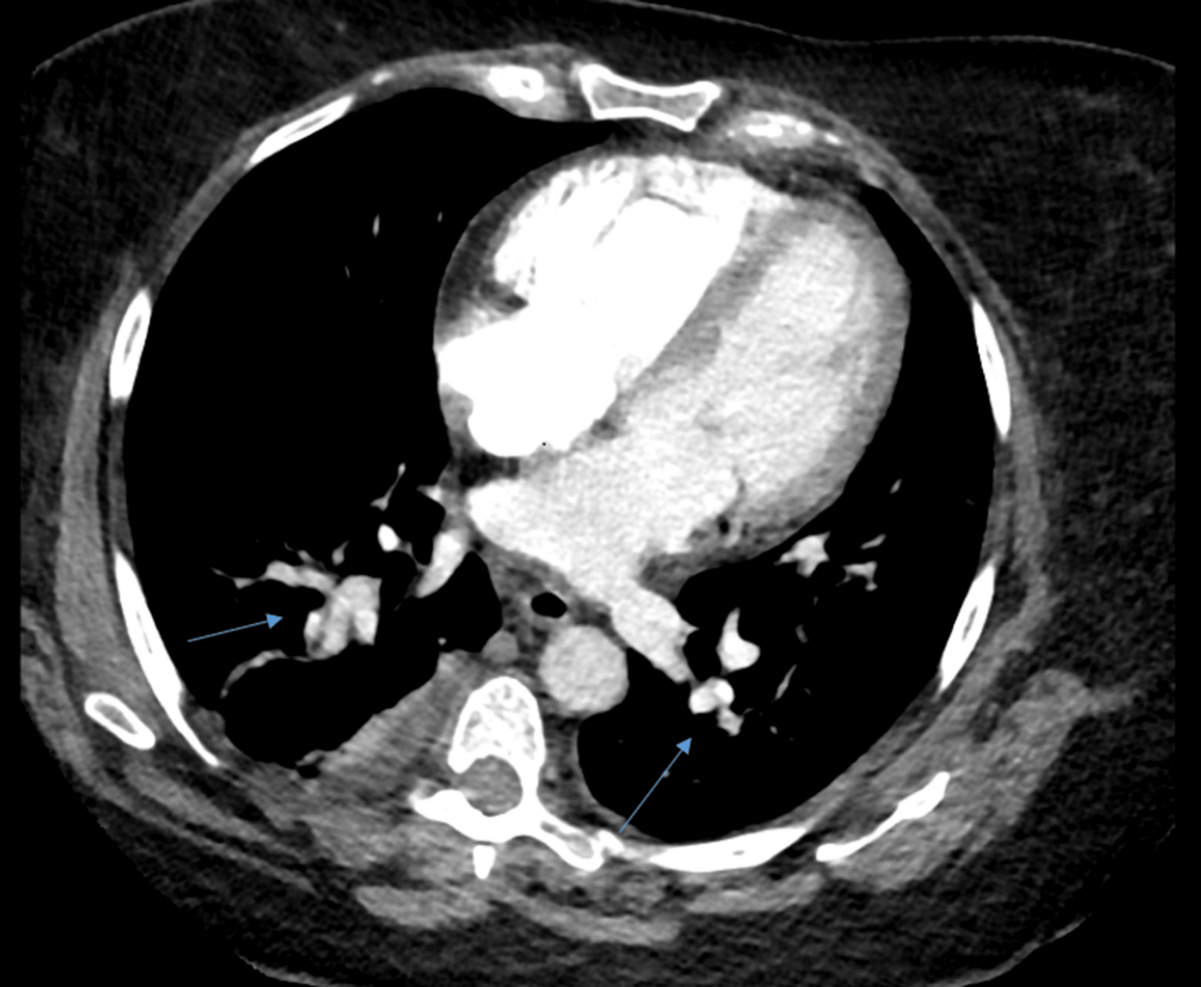
CTPA findings with blue arrows indicating the resolution of pulmonary artery thrombosis after ultrasound-assisted catheter-directed thrombolysis CTPA: computed tomography pulmonary angiography

Follow-up echocardiography on post-procedure day 1 demonstrated the normalization of RV size and systolic function, with a tricuspid annular plane systolic excursion (TAPSE) of 1.8 cm. Moderate pulmonary hypertension persisted (pulmonary artery systolic pressure (PASP): 53 mmHg). Four days later, repeat CTA revealed the near-complete resolution of the pulmonary emboli with only faint residual thrombi (Figure 2). The patient showed significant clinical improvement and was discharged on oral anticoagulation therapy.

Case 2 

A 29-year-old woman with a prior history of PE two years earlier presented with a two-day history of retrosternal pleuritic chest pain, progressively worsening dyspnea, bilateral leg swelling, and dry cough. She denied fever, hemoptysis, or trauma. On examination, she was hemodynamically stable but desaturated to 88% on room air and exhibited tachypnea and tachycardia. Left calf swelling was noted.

**Figure 3:**
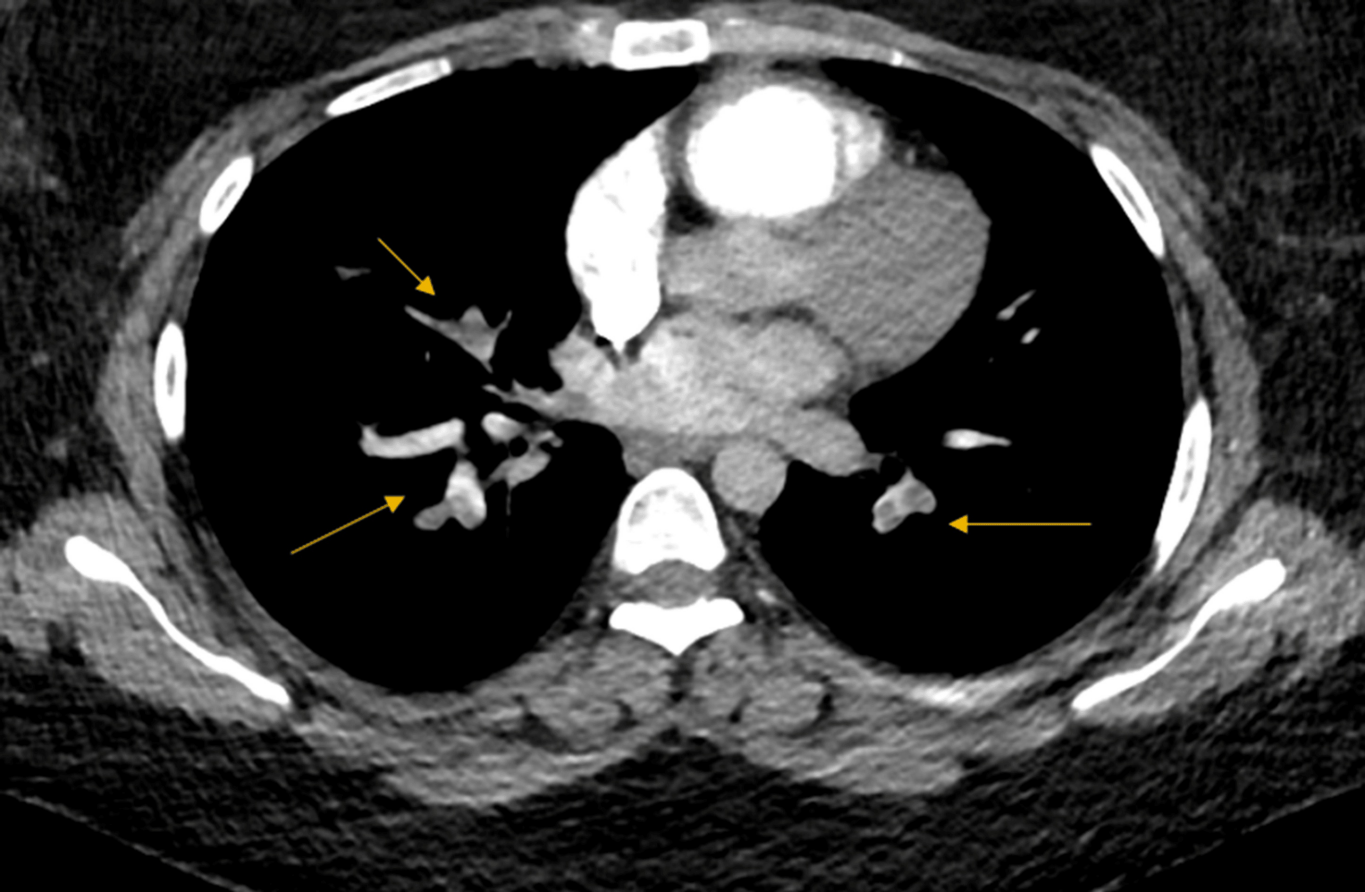
CTPA showing multiple bilateral lobar and segmental pulmonary emboli (yellow arrows) CTPA: computed tomography pulmonary angiography

The Wells score was 9. Laboratory workup showed elevated troponin (1.416 µg/L), BNP (44 pmol/L), and D-dimer (7.1 µg/mL). TTE revealed a mildly dilated RV with signs of RV strain (RV/left ventricular (LV) ratio >1), estimated RV systolic pressure (RVSP) of 25 mmHg, and echocardiographic signs of PE. CTA confirmed multiple bilateral lobar and segmental pulmonary emboli (Figure 3). Venous duplex ultrasound demonstrated a partially occlusive DVT in the left lower limb extending to the distal segment of the superficial femoral vein.

Due to the high-risk classification on PESI, the patient underwent UCDT without complications. Follow-up TTE on day 1 post-procedure showed the normalization of RV size and function with no evidence of pulmonary hypertension. A follow-up CTA was not performed per the patient's preference. She was discharged on anticoagulation therapy with a favorable clinical outcome.

Case 3

A 69-year-old woman with a history of obesity hypoventilation syndrome, chronic obstructive pulmonary disease (COPD) on home bilevel positive airway pressure (BiPAP), and morbid obesity (BMI: 54) presented with dyspnea, palpitations, and left lower limb pain. On examination, she was hemodynamically stable but desaturated to 88% on room air, with tachypnea and tachycardia. Left calf swelling was also noted.

**Figure 4:**
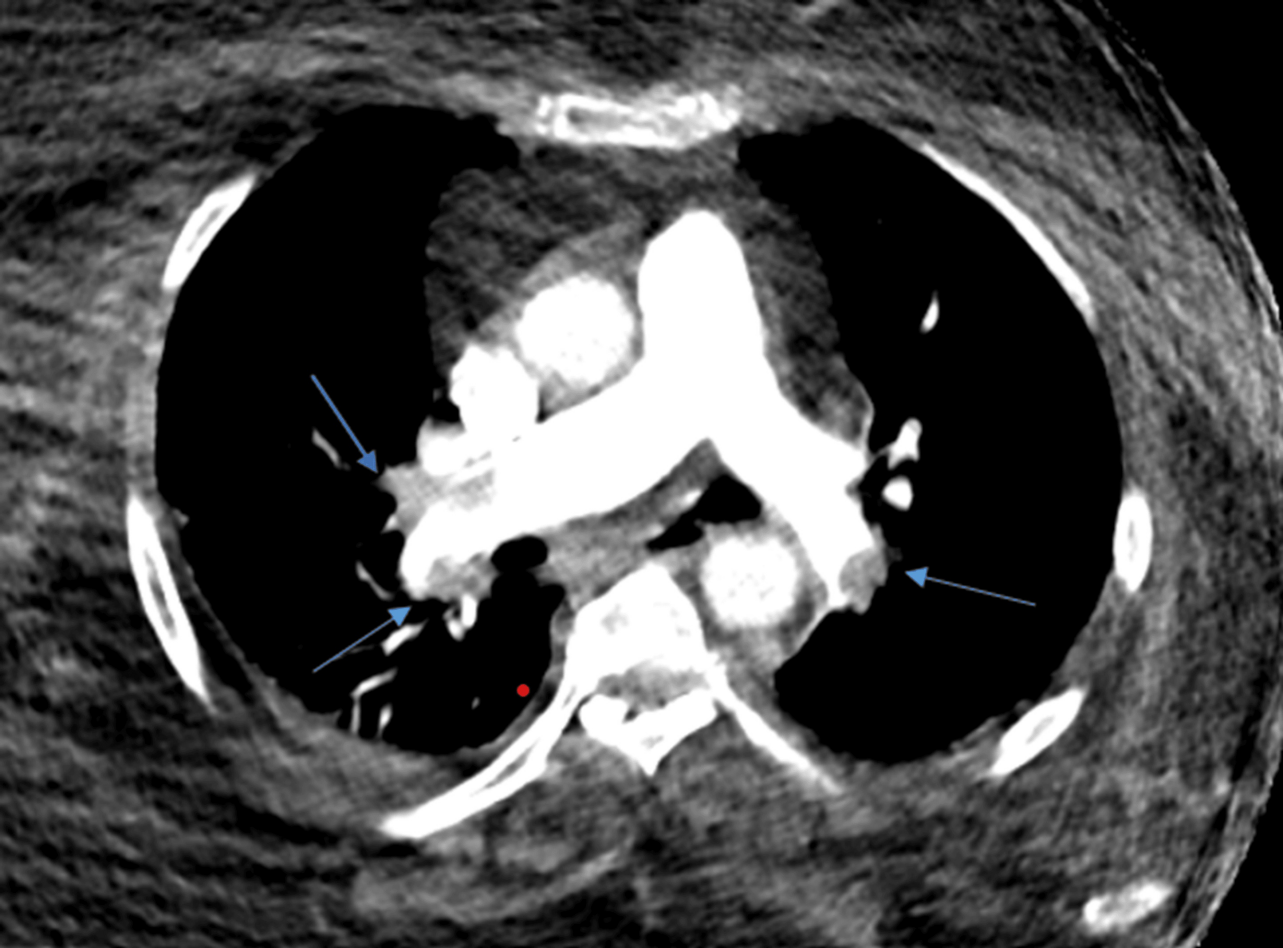
CTPA confirming bilateral acute pulmonary embolism (blue arrows) CTPA: computed tomography pulmonary angiography

The Wells score was 7.5. Laboratory results showed elevated troponin (0.031 µg/L), BNP (115 pmol/L), and D-dimer (2.71 µg/mL). TTE findings were consistent with acute massive or submassive PE, with a D-shaped LV, RV/LV ratio >1, TAPSE of 1.5 cm, and McConnell's sign. Urgent CTA confirmed bilateral acute PE (Figure 4). Venous duplex ultrasound revealed extensive left lower limb DVT extending from the popliteal vein to the femoral vein, terminating just before the common femoral vein.

**Figure 5:**
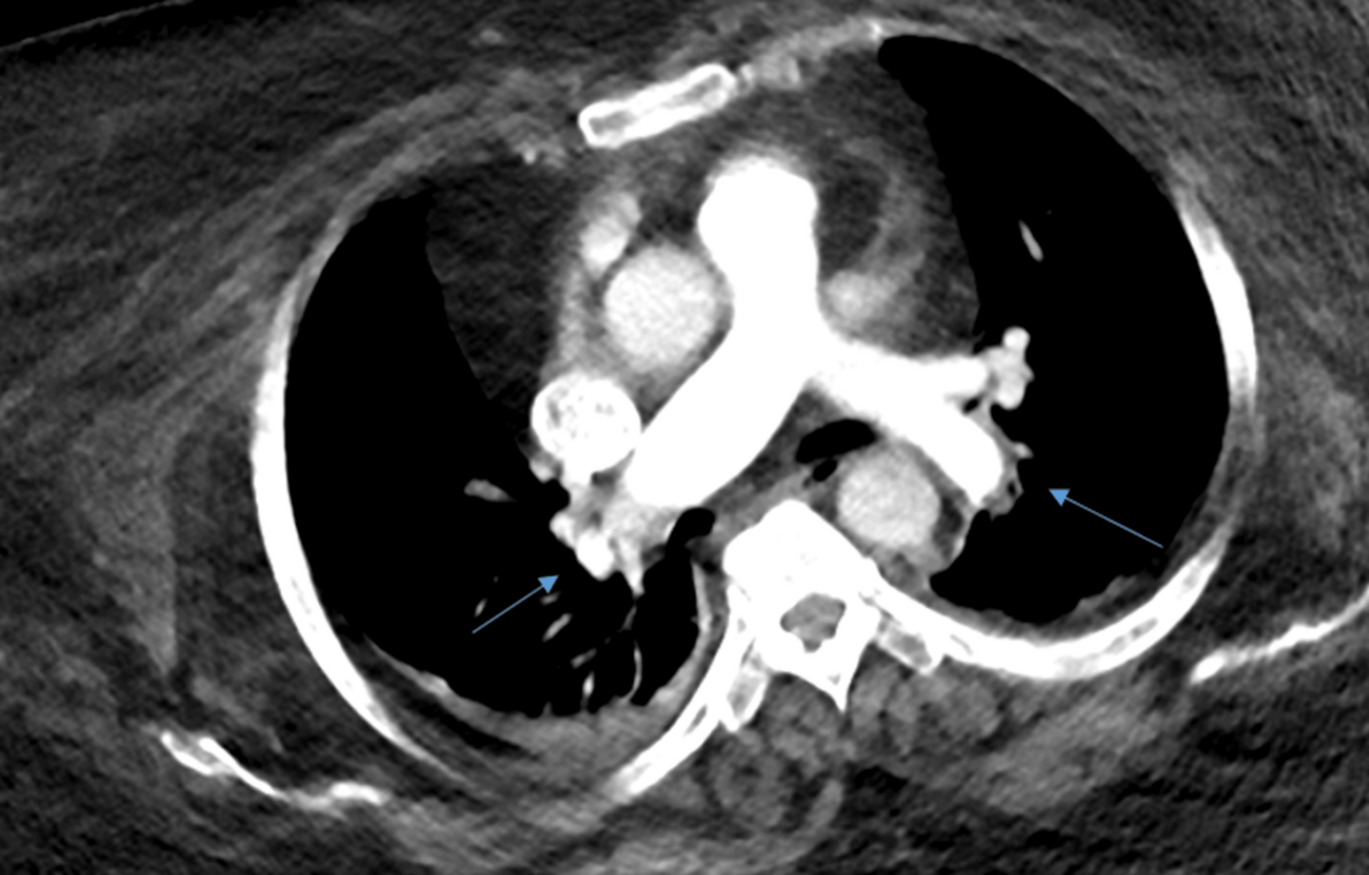
CTPA showing the resolution of pulmonary embolism with blue arrows indicating areas of reperfusion after ultrasound-assisted catheter-directed thrombolysis CTPA: computed tomography pulmonary angiography

Initially, the patient was managed with therapeutic anticoagulation and close monitoring. However, 48 hours later, her condition deteriorated. Given the high-intermediate risk classification, UCDT was performed. Post-procedure echocardiography on day 1 showed marked improvement in RV size and function, with an RV/LV ratio <1, the resolution of McConnell's sign, and an increase in pulmonary valve acceleration time (from 45 ms pre-procedure to 76 ms post-procedure). Follow-up CTA five days later confirmed the significant resolution of the pulmonary emboli (Figure 5). The patient had an uneventful recovery and was discharged with continued anticoagulation therapy.

Table 1 summarizes the baseline vitals and laboratory investigations of the patients, highlighting key hemodynamic parameters and biomarkers relevant to PE severity and management with UCDT.

**Table 1:** Baseline vitals and laboratory findings of the patients undergoing ultrasound-assisted catheter-directed thrombolysis for pulmonary embolism BNP: B-type natriuretic peptide

Parameter	Patient 1	Patient 2	Patient 3	Reference range
Heart rate (bpm)	120	110	115	60-100
Blood pressure (mmHg)	70/40	85/50	90/60	90-120/60-80
Oxygen saturation (%)	88	90	89	>95
Respiratory rate (breaths/min)	28	25	27	12-20
D-dimer (µg/mL)	3.32	7.1	2.71	<0.5
Troponin (µg/L)	1.48	1.416	0.031	<0.04
BNP (pmol/L)	64.5	44	115	<100

## Discussion

PE is a significant cause of morbidity and mortality worldwide. Traditional management includes systemic anticoagulation and, in severe cases, systemic thrombolysis [1]. However, systemic thrombolysis carries substantial bleeding risks, prompting the exploration of alternative therapies [2]. UCDT has emerged as a promising intervention, aiming to enhance thrombolytic efficacy while minimizing hemorrhagic complications [3].

Effective management of PE necessitates accurate risk stratification to guide therapeutic decisions. Patients are typically categorized into low-, intermediate-, and high-risk groups based on clinical parameters, imaging findings, and biomarkers [4]. High-risk PE, characterized by hemodynamic instability, often warrants aggressive interventions such as systemic thrombolysis or surgical embolectomy [5]. However, the substantial bleeding risk associated with systemic thrombolysis has led to the consideration of catheter-directed therapies [6]. The American Society of Hematology (ASH) guidelines suggest that for patients with acute PE and evidence of RV dysfunction, anticoagulation alone is preferred over the routine use of thrombolysis [7]. Thrombolysis may be considered for patients at low bleeding risk who are at high risk for decompensation. In this context, UCDT offers a targeted approach, delivering thrombolytic agents directly to the clot, potentially reducing systemic exposure and associated risks [8].

Systemic thrombolysis is the standard therapy for acute massive PE; however, it carries an estimated 20% risk of major hemorrhage, including a 3-5% risk of hemorrhagic stroke [5]. Catheter-directed thrombolysis (CDT) offers targeted clot dissolution, reducing systemic exposure and potentially decreasing bleeding risks [9]. UCDT further enhances this approach by using ultrasound energy to facilitate deeper thrombolytic penetration into the thrombus [6]. Current guidelines suggest that CDT may be considered in patients with high-risk PE who have high bleeding risk, after failed systemic thrombolysis, or in patients with rapid hemodynamic deterioration as a bail-out before systemic thrombolysis can be effective [10].

In our case series, three patients with high-risk and intermediate-high-risk PE underwent UCDT. All patients demonstrated significant improvements in RV function and hemodynamic stability post-procedure, with no major complications reported. These findings are consistent with the ULTIMA trial, which showed that UCDT is superior to anticoagulation with heparin alone in reversing RV dilatation at 24 hours, without an increase in bleeding complications [1].

A meta-analysis evaluating UCDT in acute PE patients reported significant improvements in hemodynamic parameters and a favorable safety profile [2]. Additionally, a nationwide inpatient cohort study found that among patients with shock, CDT was associated with lower in-hospital mortality compared to systemic thrombolysis [9]. These studies support the efficacy and safety of UCDT in managing high-risk and intermediate-high-risk PE [10].

UCDT offers a viable alternative for PE patients, particularly those with contraindications to systemic thrombolysis or those at high risk of bleeding [8]. Its targeted approach allows for effective clot resolution with reduced thrombolytic dosages, potentially minimizing bleeding complications [7]. Implementing UCDT may also lead to shorter intensive care unit stays and improved overall patient outcomes [3]. However, patient selection is crucial, and a multidisciplinary team approach is recommended to optimize treatment strategies [4].

Our case series is limited by its small sample size and lack of long-term follow-up, which may affect the generalizability of the findings [5]. Future research should focus on large-scale, randomized controlled trials to further evaluate the efficacy and safety of UCDT, refine patient selection criteria, and establish standardized treatment protocols [6]. The ongoing Higher-Risk Pulmonary Embolism Thrombolysis (HI-PEITHO) trial aims to establish the first-line treatment in intermediate-high-risk PE patients with imminent hemodynamic collapse and is expected to inform international guidelines [10].

## Conclusions

This case series highlights the effectiveness of UCDT in improving hemodynamic stability and RV function in high-risk and intermediate-high-risk PE. All patients demonstrated significant clinical and echocardiographic improvements without major complications. The findings suggest that UCDT offers a safer and effective alternative to systemic thrombolysis, particularly for patients at high risk of bleeding. While this technique appears promising, further large-scale studies are necessary to confirm its long-term efficacy and establish standardized treatment protocols.

## References

[1] Kucher N, Boekstegers P, Müller OJ (2014). Randomized, controlled trial of ultrasound-assisted catheter-directed thrombolysis for acute intermediate-risk pulmonary embolism. Circulation.

[2] Kaymaz C, Akbal OY, Tanboga IH (2018). Ultrasound-assisted catheter-directed thrombolysis in high-risk and intermediate-high-risk pulmonary embolism: a meta-analysis. Curr Vasc Pharmacol.

[3] Al-Terki H, Mügge A, Gotzmann M (2023). The safety and efficacy of ultrasound-accelerated catheter-directed thrombolysis in patients with intermediate-high-risk pulmonary embolism: Bo-NE-Experience. J Clin Med.

[4] Konstantinides SV, Meyer G, Becattini C (2020). 2019 ESC guidelines for the diagnosis and management of acute pulmonary embolism developed in collaboration with the European Respiratory Society (ERS). Eur Heart J.

[5] Chatterjee S, Chakraborty A, Weinberg I (2014). Thrombolysis for pulmonary embolism and risk of all-cause mortality, major bleeding, and intracranial hemorrhage: a meta-analysis. JAMA.

[6] Zbinden S, Voci D, Grigorean A (2023). Clinical outcomes of ultrasound-assisted coagulation monitoring-adjusted catheter-directed thrombolysis for acute pulmonary embolism. Thromb Res.

[7] Piazza G, Hohlfelder B, Jaff MR (2015). A prospective, single-arm, multicenter trial of ultrasound-facilitated, catheter-directed, low-dose fibrinolysis for acute massive and submassive pulmonary embolism: the SEATTLE II study. JACC Cardiovasc Interv.

[8] Vedantham S, Goldhaber SZ, Julian JA (2017). Pharmacomechanical catheter-directed thrombolysis for deep-vein thrombosis. N Engl J Med.

[9] Voci D, Zbinden S, Micieli E, Kucher N, Barco S (2022). Fixed-dose ultrasound-assisted catheter-directed thrombolysis for acute pulmonary embolism associated with COVID-19. Viruses.

[10] Tapson VF, Sterling K, Jones N (2018). A randomized trial of the optimum duration of acoustic pulse thrombolysis procedure in acute intermediate-risk pulmonary embolism: the OPTALYSE PE trial. JACC Cardiovasc Interv.

